# A user‐centered, learning asthma smartphone application for patients and providers

**DOI:** 10.1002/lrh2.10217

**Published:** 2020-02-18

**Authors:** Mark Gaynor, David Schneider, Margo Seltzer, Erica Crannage, Mary Lee Barron, Jason Waterman, Andrew Oberle

**Affiliations:** ^1^ Saint Louis University (SLU) College for Public Health and Social Justice (CPHSJ) St. Louis Missouri; ^2^ University of Texas Southwestern Medical Center Dallas Texas; ^3^ University of British Columbia Vancouver Bristish Columbia Canada; ^4^ Saint Louis College of Pharmacy St. Louis Missouri; ^5^ Southern Illinois University Carbondale Illinois; ^6^ Vassar College Poughkeepsie New York

**Keywords:** asthma, health care, intelligent system, self‐management, smartphone application, user‐centered

## Abstract

**Problem:**

Smartphone applications are an increasingly useful part of patients' self‐management of chronic health conditions. Asthma is a common chronic health condition for which good self‐management by patients is very helpful in maintaining stability. User‐centered design and intelligent systems that learn are steps forward in building applications that are more effective in providing quality care that is scalable and tailored to each patient.

**Methods:**

A literature and application store search to review historic and current asthma smart phone applications. User‐centered design is a methodology that involves all stakeholders of a proposed system from the beginning of the design phase to the end of installation. One aspect of this user‐centered approach involved conducting focus groups with patients and health care providers to determine what features they desire for use in applications and create a model to build smart infrastructure for a learning health care system. A simple prototype for an asthma smartphone application is designed and built with basic functionality.

**Outcomes:**

Only one publication in the literature review of asthma smartphone applications describes both user‐centered design and intelligent learning systems. The authors have presented a set of user‐desired attributes for a smart health care application and a possible data flow diagram of information for a learning system. A prototype simple user‐centered designed asthma smartphone application that better assists patients in their care illustrates the value of the proposed architecture.

**Discussion:**

Our user‐centered approach helped design and implement a learning prototype smart phone application to help patients better manage their asthma and provide information to clinical care providers. While popular in other industries, user‐centered design has had slow adoption in the health care area. However, the popularity of this approach is increasing and will hopefully result in mobile application that better meets the needs of both patients and their care providers.

## 1 INTRODUCTION

The use of smartphones has evolved from mere usage for communication to manage one's own lifestyle, including one's health. Certain features of health‐related applications have been found to have a more profound effect on changing the patient's habits.^1^, ^2^, ^3^, ^4^, ^5^, ^6^, ^7^, ^8^, ^9^ However, many health care smartphone applications do not learn and are not designed with a user‐centered design (UCD) approach. Because of this, these applications can be difficult to use and may have limited utility. The concept of UCD became popular with the publication of Donald Norman's book “A User‐Centered System Design: New Perspectives on Human‐Computer Interaction”^10^ and the revised “The Design of Everyday Things.”^11^ UCD provides a framework in which all stakeholders of a system help define the goals and features of a system. This includes interaction between designers, developers, and all users. Focus groups are conducted to determine the needs and goals of the stakeholders. These ideas are used to create simple prototypes that are tested with users, which subsequently develop into more complex, interactive prototypes that allow the features and functions of a system to be iteratively tested and evolve to best meet the needs of the stakeholders.

This user centric methodology is likely to produce systems that have high usability by using a general process that includes:

- Identifying the users of a system and what they want.
- Define requirements from the users' needs defined in step a.
- Create iterative designs with user feedback.
- Evaluate the final design.

The methodology to develop a persuasive and long‐term sticky application is driven by users from the very beginning with UCD principles that focus on understanding the user and building the users' mental model into the functional system. This UCD methodology is not a commonplace in health care applications, including self‐management of chronic conditions. When this study was started, it was one of the first studies of its kind in the area of asthma applications with a UCD approach that includes both patients and providers within the focus groups; however, more current research has adopted this approach.^12^ This two‐prong approach allows for a more complete understanding of what patients and providers both desire in a smartphone application for the self‐management of health conditions. By creating an application from the data collected by a UCD, the user will have a better experience. Recent studies that have applied UCD to redefining interfaces in the health care setting show significant improvements in system usefulness, information quality, and interface quality. UCD is well recognized as an effective strategy for designing ease‐of‐use into the total customer experience with products and systems and has been used to design interfaces to medical devices.^1^, ^2^, ^3^, ^4^, ^6^, ^7^, ^8^, ^9^, ^13^, ^14^, ^15^ Evidence has shown that IT projects that fail to incorporate input from users are more likely to not meet their needs. One good example for health care is the implementation of an Electronic Health Records (EHRs) system at Cedars‐Sinai in Los Angeles.^16^ Users were not properly included in the system design, and the clinical care providers revolted, refusing to adopt the system.

Another lacking feature of these applications is the absence of an intelligent infrastructure that promotes smart learning and tailored customization. Current asthma applications do not fully utilize innovative technology available in the market, such as automatic integration of data from peak flow meters and connection with EHRs. Self‐management asthma applications are not effective for patients or health care providers because key features, such as device integration, Artificial Intelligence (AI),^17^ customization, and logging mechanisms for data collection for public health purposes and personal health records, are not combined. These key features are missing because patients, health care providers, and the community are not typically involved with the design and implementation of smartphone applications.

One possible feature to increase the patient's compliance with medication for asthma is a notification system that sends patients an alert or a text through the application to remind the patients to take their medicine.^18^ Pharmacists believe that patients utilizing these self‐management applications would show an increase in medication adherence. Nationwide Children's has recently developed the first mobile application shown to be effective in a clinical study to provide education, improve the use of controller medication, and teach self‐management of asthma to prevent attacks.^19^

Patients in contact with their primary care physician via texting were shown to increase communication with their health care provider. To have real‐time texting communication with a health care professional could be an integral aspect to assess health behavior.^20^ However, most payment systems do not provide a mechanism for health care providers to be paid for such a service, disincentivizing them from participating. By standardizing applications, it would allow for the freedom of greater device integration, which would allow more patients and providers to utilize the service.^21^

Fortunately, the development of health‐related smartphone applications is changing, as health care researchers are starting to embrace user‐centered methodologies^1^, ^2^, ^3^, ^4^, ^5^, ^6^, ^7^, ^8^, ^9^ and intelligent learning systems.^22^, ^23^, ^24^, ^25^, ^26^, ^27^, ^28^ Morrison and colleagues^29^ state that smartphone applications for individuals with asthma will more effectively aid self‐management of the condition if they are designed in collaboration with end‐user patients and utilize more sophisticated and interactive systems that continuously provide users with directed and clinically appropriate advice. Therefore, the design of asthma self‐management smartphone applications needs a user‐centered and smart learning focus because present applications are not meeting the needs of many patients or health care providers.^30^

This paper aims to address these evolving needs. We will first discuss the current literature that evaluated smartphone applications for self‐monitoring of chronic conditions that include UCD and intelligent learning systems. The paper will then discuss the authors' approach to UCD and intelligent learning systems, including the facilitation of focus groups and the evaluation of data collected from these patient and health care provider groups. A framework is then proposed for a smart learning system based on the results from our focus group. This framework includes describing a possible data flow to implement this smart health care architecture. Finally, the prototype application will be explained in detail. The prototype needs further development and testing by patients and health care providers to truly test the application's effectiveness.

## 2 METHODS

To improve the design of smart self‐management asthma applications, a UCD methodology was used that includes understanding the current literature, a review of historic and current asthma smart phone applications, focus groups of both asthma patients and health care workers, and development of a simple working prototype.

### 2.1 Literature and asthma application review

There have been many systematic literature reviews of smartphone and/or mobile applications for individuals with asthma and other pulmonary related diseases.^5^, ^31^, ^32^, ^33^, ^34^, ^35^, ^36^, ^37^, ^38^, ^39^ However, none specifically review the prevalence of applications that are designed with both user‐centered methodology and intelligent learning systems to continuously improve the user experience.

The PubMed database was searched to identify relevant publications that range from the year 2000 to 2019. Articles were included in the review if the title or abstract mentioned the phrase “asthma smartphone application” and at least one of the following keywords for UCD development: “development,” “design,” “user‐centered,” “user‐driven,” “patient‐centered”; or for intelligent learning systems: “intelligent,” “learning,” “self‐“management,” “interactive,” “patient engagement,” “smart,” and “semi‐automated.” The full text of the included articles was then reviewed to elucidate whether application developers used a UCD methodology or incorporated intelligent learning systems, both described above. Publications that were included based on a full text review were then evaluated for a focus on both explored themes.

Smartphone applications containing the term “asthma” were searched for in the Apple App Store for iPhones, Google Play Store for Android phones, and reviewed for application features.

### 2.2 User‐centered design

Our UCD approach to development starts with understanding the user and user requirements gathered directly from intended users (asthma patients) and other stakeholders (medical professionals, parents, and peers) with whom users interact.^1^, ^2^, ^3^, ^4^, ^6^, ^7^, ^8^, ^9^, ^13^, ^14^, ^15^ To understand who the users are, their goals, motivations, characteristics, application environment, and constraints, we conducted two sets of focus groups: patients and clinical care providers. This enabled an understanding of clients' medical needs, lifestyle, current coping tasks, technology perceptions and preferences, and interface preferences. In addition, patients and care providers helped create early “paper‐and‐pencil” interface prototypes and participated in usability testing of subsequent, more advanced prototypes.

**Figure 1:**
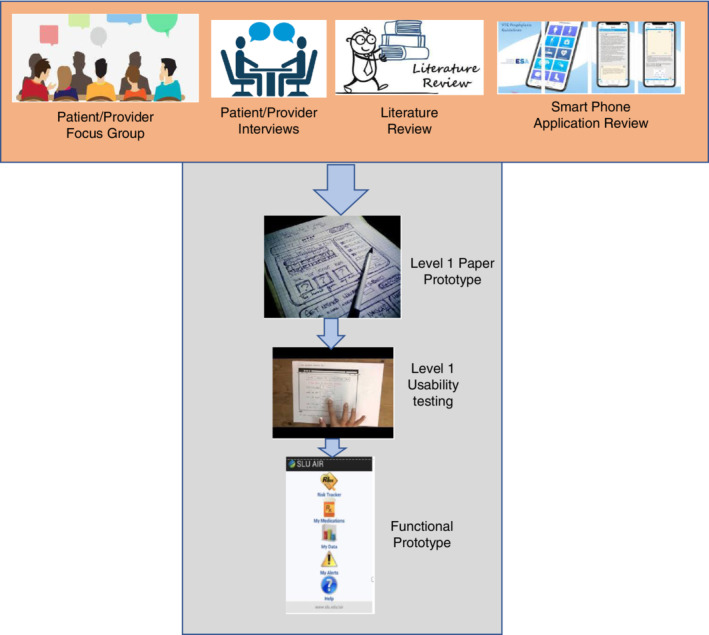
User‐centered design

To accurately meet the desires of both patients and health care providers for asthma mobile applications, a UCD was used to improve upon current asthma applications. Krueger^40^ recommends starting with three or four focus groups and evaluating the results to see if saturation has occurred. A saturation limit is achieved when new ideas are not being generated by additional focus groups. Because of our limited resources, our goal was not to reach saturation with the focus groups. Instead, we used other well‐established qualitative research tools with several formal best practices.^41^ Some applications of focus groups require saturation because the focus group is only used in a single point in the process to address the needs of the focus group members. With our iterative approach to UCD, there are several points in the development process to incorporate new ideas, such as in the paper‐drawn prototypes and the developed simple working prototype (Figure 1). Direct observation of the patient, which is an important part of UCD, was not included at this early stage. The research team believes this observation is of more value if accomplished with the functional prototype.

After the focus groups met, the research assistant (RA) coded the focus group conversations using traditional qualitative techniques where data were coded by reviewing the recorded conversations and pulling out the details of features desired by both patients and care providers.

## 3 RESULTS

### 3.1 Literature and asthma application review

**Figure 2:**
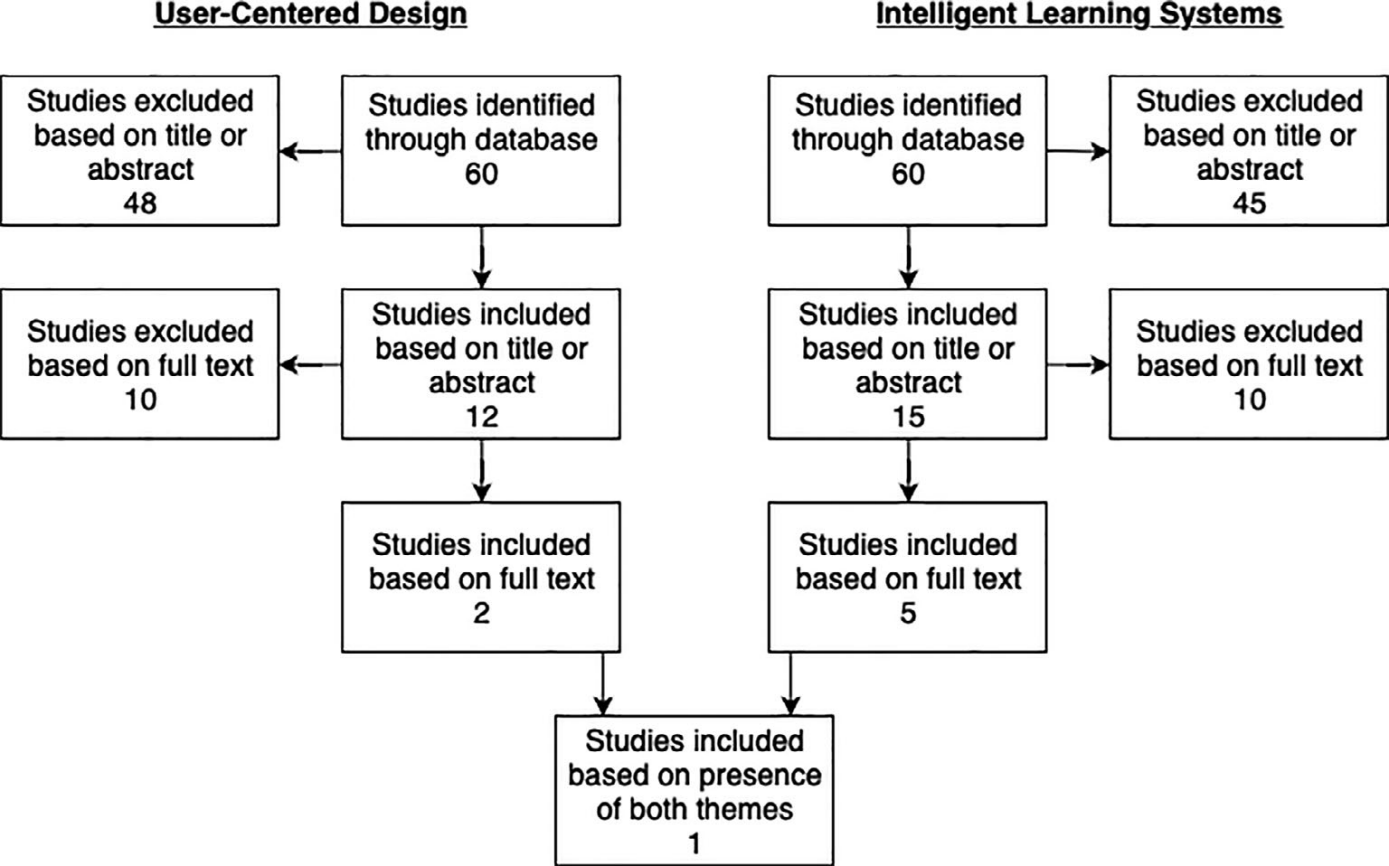
Literature review flow diagram

A PubMed search of the term “asthma smartphone application” resulted in 60 academic articles, all of which were published between 2012 and 2019. Each resulting publication was then reviewed for UCD methodology and an intelligent learning system in the development of the reported application (Figure 2).

A review of the 60 asthma smartphone application articles for UCD principles resulted in the exclusion of 48 because their titles or abstracts did not mention the development of the smartphone application or mention engaging patient end users in the development of the smartphone application. Of the remaining 12, only two described a user‐centered approach during application development.^28^, ^42^ Of the excluded articles, three mentioned engaging patients with asthma by having them test and evaluate prototype applications but made no mention of incorporating patient feedback into further development of the application.^12^, ^43^, ^44^ A review of the 60 articles for intelligent learning system principles resulted in the exclusion of 45 articles because the title or abstract did not include any of the keywords to indicate the presence of an intelligent learning system. The full text of remaining 15 articles was then read to determine if the application included an intelligent system that could learn and continuously improve usability for the patient. Only five of the remaining 15 articles discuss the presence of such a system.^24^, ^27^, ^28^, ^45^ Of the original 60 articles, only one described using both a UCD and an intelligent learning system in the application's development.^28^

By searching the word “asthma” in the Apple App Store and the Google Play Store, hundreds of applications appear. These applications have an array of uses for patients, such as journaling, education, setting medication alarms, or even communicating with the patient's primary care provider.^20^ There are many effective features from different applications, such as customization, standardization, communication with a health care provider, and notifications to increase compliance of medication (Tables 1 and 2). However, smartphones are not being fully utilized as current applications are lacking the combination of key features that patients need to manage their asthma.^5^, ^29^ Smartphones have the multifaceted capability sought, but as seen in Tables 1 and 2, a properly designed application is needed to fit the needs of both the patient and the provider.^30^

**Table 1:** Review of asthma applications iOS and Android

	App																
Feature	SLU AIR	1	2	3	4	5	6	7	8	9	10	11	12	13	14	15	16
Daily Journal	✓	✓	✓	✓		✓			✓	✓	✓	✓	✓	✓		✓	✓
Graph or Chart	✓	✓	✓	✓		✓		✓	✓	✓	✓	✓	✓	✓		✓	✓
Peak Flow	✓	✓	✓	✓		✓			✓	✓	✓			✓		✓	
Asthma Zones Green, Yellow, Red	✓	✓	✓	✓		✓				✓		✓		✓		✓	
Track Triggers	✓	✓	✓					✓			✓		✓				✓
Location	✓	✓									✓		✓				
Forced expiratory volume in 1 second	✓																
Export Data	✓	✓	✓					✓	✓	✓	✓	✓	✓	✓		✓	
Emergency Alert	✓	✓			✓												
Action Plan	✓	✓	✓					✓			✓						✓
Symptoms	✓	✓	✓						✓	✓		✓			✓		✓
Free	✓	✓	✓	□	✓	✓					✓	✓	✓	✓	✓	✓	✓
Prescription Tracker	✓	✓	✓	✓	✓	✓		✓	✓	✓	✓	✓	✓			✓	✓
Educational	✓	✓					✓	✓				✓					✓

**Table 2:** Application names and descriptions used in Table 1

App #	Name	Description	Platform
1	Propeller Health^46^	Asthma and COPD management; geospatial tracker, planner, and trigger identification; patient‐provider communication (FDA‐cleared, sensor‐based)	iOS, Android
2	Asthma MD^47^	Track and research asthma	
3	asthmaTrack^48^	Record symptoms, treatments, vital signs, and environmental conditions using pre‐loaded templates	iOS only
4	ASTHMA^49^	Medication reminder and emergency assistance	iOS only
5	Asthma Tracker^50^	Track asthma peak flow, medication, and symptoms	iOS, Android
6	Asthma+^51^	Asthma education	iOS only
7	Asthma Action Hero^52^	Interactive asthma action planning and tracker; education (pediatric)	iOS, Android
8	Asthma‐Diary^53^	Asthma symptom and medication tracker	iOS only
9	Asthma Manager^54^	Asthma symptom tracker and medication management; patient‐provider communication; education	iOS only
10	SaniQ Asthma^55^	Asthma symptom and medication tracker and management; reminders; environmental notices	iOS, Android
11	My Asthma Pal^56^	Asthma symptom and medication tracker; education	iOS, Android
12	FindAir^57^	Asthma symptom and medication tracker and management; reminders; environmental notices	Android only
13	Peak Flow^58^	Peak flow tracker	Android only
14	Asthma Test^59^	Asthma tracker	Android only
15	PEF Log‐asthma tracker^60^	Asthma tracker	iOS, Android
16	rg breathe^61^	Asthma symptom management and tracker	Android only

### 3.2 Focus group

The focus group used in the UCD methodology illustrated in Figure 1 determined which features both patients and providers desire in a smartphone application to manage asthma. Design requirement focus groups defined the basic application functional requirements that will be most effective and persuasive for our asthma self‐management application. The focus group data reflected that patients and providers sought customizations of the application at a low cost to the patient. These customizations include environmental trigger warnings, education on asthma for the patient, the ability to contact the pharmacy, a list of emergency contacts, the ability to contact health care providers, the interoperability of connecting to the patient's EHR, medication and appointment trackers, a log for symptoms, and integration of a peak flow meter with Bluetooth information to create a graph of these data.

Two focus groups of eight asthmatic patients and two focus groups of eight health care providers were conducted to better understand the desired requirements of self‐management smartphone applications for asthmatics. The members of these focus groups consisted of the authors' personal contacts and patients of other authors. Several authors, who are clinical care providers, were members of the provider focus groups. All members of the focus group were above the age of 18. While some participants may have had previous experience with self‐management applications, they were not asked to research or use asthma applications before the focus group. The groups of care providers included several nurses and family physicians, a pharmacist, and a pulmonologist. Their experience ranged from several years to several decades with asthma patients. The pharmacist in the provider group is also an asthma educator. The patient groups varied demographically, from younger very active to less active individuals. During the focus groups, cohorts worked together to create their ideal self‐management application for asthma. Features important to both groups are described in Table 3. There are several differences between attributes that patients and their clinical care provider desire. Patients are interested in what effects them such as low cost and emergency contacts. Care providers are more interested in features related to patient monitoring such as connection to the EHR, automatic inclusion of peak flow measurements into the EHR, and the ability to connect with the pharmacy.

**Table 3:** Asthma application features requested from focus group participants

Quality Sought in an Application	Health Care Providers	Asthma Patients
Customization	✓	✓
Low Cost		✓
Tracking of Environmental Triggers	✓	✓
Education	✓	✓
Ability to Contact Pharmacy	✓	
List Emergency Contacts		✓
Ability to Communicate between Patient and Provider	✓	✓
Ability to Connect to Medical Record	✓	
Medication Tracker	✓	✓
Symptoms Journal	✓	
Device Integration	✓	✓
Chart of Peak Flow Log	✓	
Notification system	✓	✓

Our prototype application incorporates some of the requested features discovered in the focus groups. Several features, such as customization, education, environmental triggers, device integration, the ability to contact the health care provider, and a medication tracker, are sought by both patients and health care professionals. Patients have very different lifestyles and needs. One member in a patient focus group was an avid runner and traveled for business while other patients did not exercise outside, or at all. This highlighted the need for customization. Knowing what triggers are currently affecting asthma patients is important to all patients, but understanding triggers across the country or world is especially critical to some. Focus group members preferred many different fitness and health monitoring devices. While patients track peak flow, weight, blood pressure, and other vital signs, there is no one device or set of standards to interface to different devices. This highlights the importance of flexibility in connecting to fitness and medical devices. Both patients and providers noted that communication with their care provider is important, and part of this communication should include a medication tracker that records maintenance and episodic medication.

Providers believe that customization is critically important because patient education is more effective when it is tailored to the specific patient. Another customization that was highlighted is the ability to track specific triggers and notification options so the patient can stay well informed. Patients also sought customization. It is important to patients to have the ability to choose to receive notifications about triggers based on the user's location, different options of what to do if it is a high‐trigger day for the user, reminders about doctors' appointments, the ability to add emergency contacts, and to receive reminders about medication.

Education was an integral aspect sought by both patients and providers for the overarching goal of educating patients to help them better self‐manage their asthma. Both patients and providers want patients to better understand causes and effects of their asthma, enabling them to control and possibly prevent asthma attacks. A feature on home remedies was desired by patients to help educate them in case they may be at a location where they do not have their medicine. Adding an educational piece on medications to help them to understand the difference between their medications and when to take them was important to providers. Overall, customization of the education to each asthma patient is ideal to assure that the patient is receiving the best information for his or her asthma.

The ability to communicate in both directions between the patient and provider was desired by both patients and providers for transparency and guidance in case of an emergency. If the provider could not be reached, the providers agreed that the application should have an asthma “action plan” that is predetermined based on a survey the patient would take in the application about their symptoms. Based on their symptoms, the application would have a preset response as to the action the patient should take in receiving medical care. For example, if the patient needed a nebulizer treatment, the patients would like the application to then give suggestions of where to go to receive the treatment and include cost transparency of the different locations.

### 3.3 Intelligent learning systems

An intelligent learning health system enables knowledge generation to be used to improve care,^62^ and our proposed infrastructure has three main components of such a system:

1. Sensing the patient and their environment, including patient vital signs, weather, or pollution.
2. Learning about the patient, including understanding what triggers a patient's asthma, what activities they do, and what their travel plans are.
3. Suggesting tailored behavior to the patient, including a caution to avoid outside activity because the pollen that triggers a patient's asthma is high in the area the patient is in at any given time.

**Figure 3:**
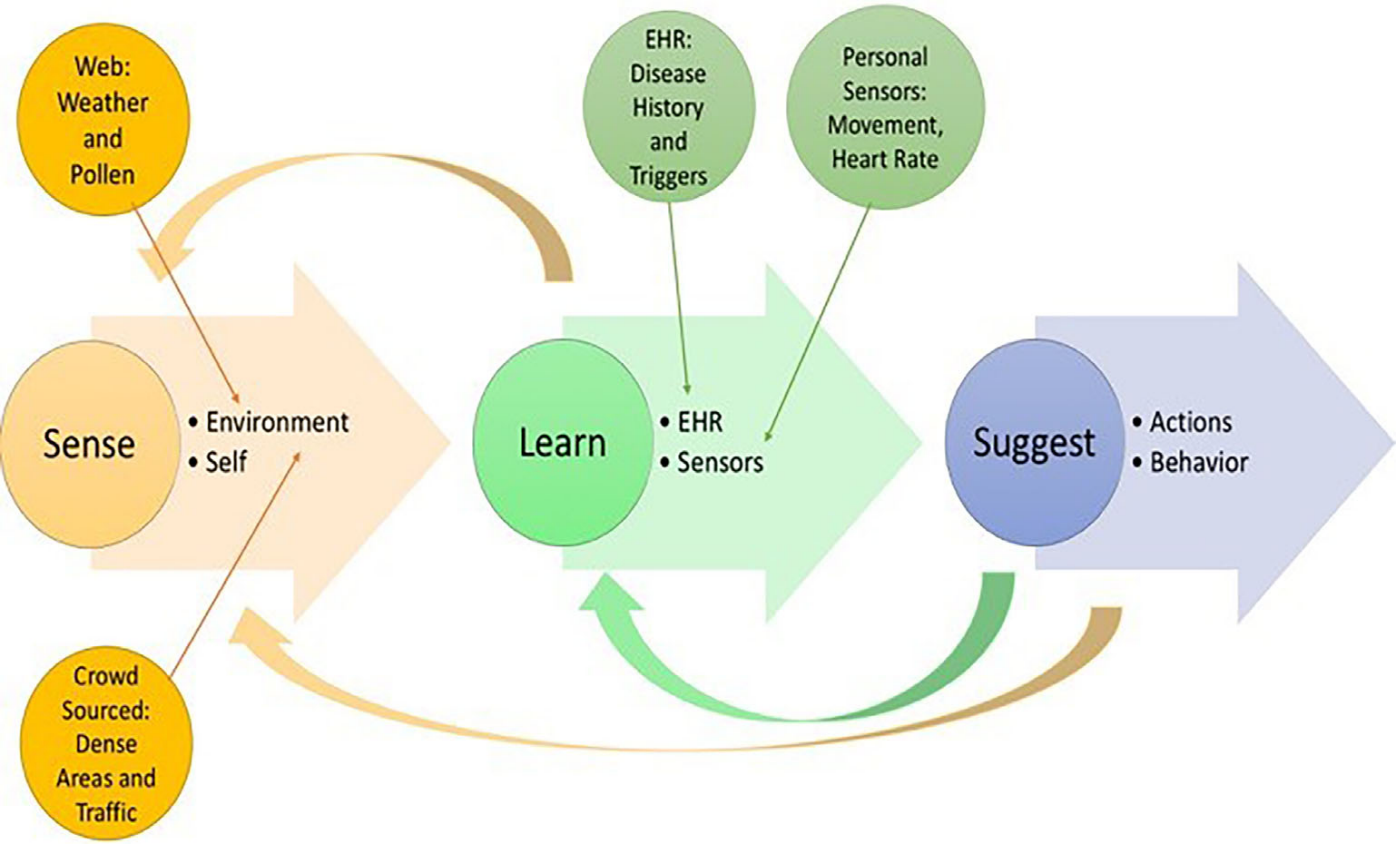
A smart system with feedback loops

Sensing, learning, and suggesting infrastructure will be able to sense the user and crowd‐sourced data, learn the user's health, and implement the use of machine learning via sensors to learn patterns and suggest a proper course of action for the user to follow. The various sources of data to sense and learn to offer suggestions can be seen in Figure 3. The three aspects of this infrastructure do not strictly follow the chain of sensing to learning to suggesting, but each aspect is able to provide feedback to the previous stage. A particular outcome of a suggestion can be used to influence further suggestions; thus, ensuring learning is affected by the suggestions. The more the system learns about the user and the environment, the better‐tailored feedback the infrastructure is able to provide. An example of this system implemented could be a patient that has asthma triggered by cottonwood pollen. They were planning a trip to a park, which publishes the pollen count on its website. The system then pulls the park's pollen count to warn the user that prior history suggests cottonwood pollen causes attacks, that there is a high pollen count, and automatically suggests they should bring their inhaler with them in case of an adverse event.

The feedback loops allow the system to continually improve itself and offer better suggestions to the users. This infrastructure can be implemented on multiple levels, from large health care systems to smart phone applications. Medical offices with this system can practice more efficiently by sensing the current patient load and learning their histories, including time‐per‐visit and chief complaint. With this information, the system could suggest to the physician the optimal order to see the patients in to provide better care at a lower cost.

**Figure 4:**
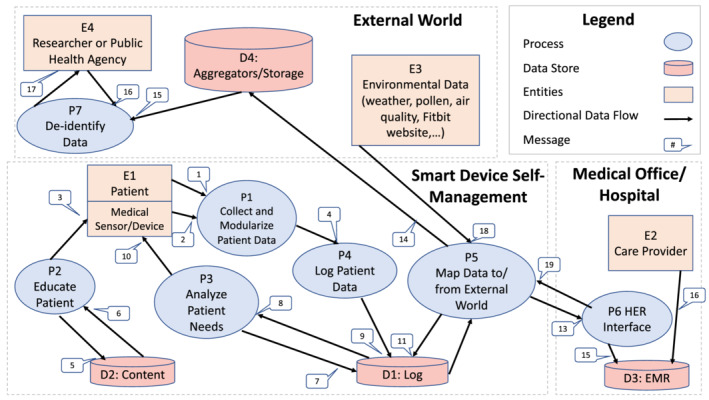
Data flow diagram

The focus groups and the authors' previous experience developing similar applications for other health‐related conditions allowed the development of a generalized approach for an asthma application to help patients self‐manage their chronic condition. Our research team developed a generalized data flow model based on the requirements defined in our focus group (illustrated below in Figure 4 and further described in Table 4). This data flow model is not particular to any condition but rather a general infrastructure that is applicable to manage any chronic health care condition. Data are collected from the patients and their medical sensors, medical devices, and activity trackers. This information is aggregated with data from the patient's EHR to capture a more complete picture of the patient's current health. Data from the outside world, such as weather, pollen counts, air quality, and special events that effect air quality (eg, fires), are also included in the data. These heterogeneous data are used to create tailored suggestions to promote self‐management of asthma (or any chronic condition) using the intelligent learning architecture. The infrastructure also enables the use of aggregated data for health care–related research, general public health reporting, and population health management. This design creates infrastructure that adapts to the patient's needs based on the most current information about the patient and their environment.

**Table 4:** Messages, processes, and databases for Figure 4

Number	Name	Description
**Entities**		
E1	Patient	Includes the patient and medical devices, sensors, and activity monitors
E2	Care Provider	This includes all the patients care providers. This provides access into the Patients EHR.
E3	Environmental Data	This includes environmental information important to asthma patients including weather, pollen counts, and air quality. This also includes access to web‐based infrastructure such as Fitbit's service that integrates into its activity monitors.
E4	Public Health/Research	This includes aggregated de‐identify data for public and other research.
**Processes**		
P1	Collect Patient Data	Collects and modularize data from the patient. This includes data from medical devices attached to the patient. It also includes data entered by the patient on a phone, tablet, or traditional computer. Note it does not include data from devices such as fitbit have a closed encrypted API and web based service to access this data (P5 collects this data.).
P2	Educate Patient	Provides tailored based education based aggregated data including current medical information from sensors, long term data based on the patient's EHR, and information from the external world such as air quality and weather.
P3	Analyze Patient Needs	This process tells P2 the needs of the patient. These needs are based on the current location, condition and behavior of the patient. For example, if cotton wood pollen is a trigger for a patient's asthma, and that patient is traveling in an area with high cotton wood pollen, then the patient is alerted to this condition.
P4	Log Patient Data	This logs data from the patient into the D1, the database containing personal patient data. This includes data directly from the patient and from the external world.
P5	External World	Maps data to/from the external world. This includes collecting weather and air quality, EHR data, and data from third party web based infrastructure such as fitbit.
P6	EHR Interface	This is the connection into the providers EHR. At this point, we expect to use FHIR to exchange data.
P7	De‐identify Data	Provides de‐identified data for research and public health agencies.
**Data Bases**		
D1	Log	Personal patient data including personal tracking (ie, activity, pulse, …), patient location, and external data such as weather and air quality.
D2	Content	A data store of content related to the patients' chronic condition (asthma in this case). This contains general data that is tailored to the patient by P2.
D3	EHR	Traditional EHR data from vendors such as Epic, Cerner, or Athena.
D4	Aggregator	A public store of de‐identified data for research and public health.
**Messages**		
M1	Patient Data	Data entered by the patient data about diet, medication, …
M2	Device Data	Automatically entered data from medical and activity tracking devices on patient.
M3	Education	Educational content tailored to each patient.
M4	Logging Data	Combined patient data for logging from Patient and External World.
M5	Q‐Education	Query tailored educational content from D2.
M6	Education Content	Tailored education content from D2 in response to M5.
M7	Q‐Local Data	Query local patient data.
M8	R‐Local Data	Patient data from D1in response to M7
M9	S‐Patient Data	Patient data for D1 from the patient.
M10	Goals/Alerts	Send tailored goals and reminders to patient.
M11	S‐E‐Patient Data	External data related to Patient.
M12	Share Patient Data	Patient data from D1 to share for research and public health.
M13	To‐EHR	Patient data for input into their EMR.
M14	To‐D4	Aggregated patient data for backup/research to D4.
M15	De‐Identify Data	De‐identified aggregated data for research.
M16	R‐De Data	Request for de‐identified data.
M17	R‐De Data	De‐identified data in response to request M16
M18	E‐Data	External data from the environment and web based services.
M19	From‐EHR	Patient data from their EHR used for tailored education and reminders.

The rest of this paper will focus on how this system is implemented in a smartphone application that can help its users control their asthma. A simple prototype application based on this generalized model is discussed in the next section.

### 3.4 Prototype application

Health care providers and patients stated that they would like to have the freedom of choice in picking Bluetooth devices such as peak flow meters, as well as choice in the type of smartphone they want to use. In other words, users want interoperability between the phone and medical devices and interoperability of the application to run on different smartphone devices. To enable this, an essential aspect that needs to be improved in asthma applications is standardization that allows for consumers and health care providers to have their choice of smartphone and Bluetooth medical devices.^21^ Creating a standardized Application Program Interface (API) that works across smartphone platforms (ie, iPhone and Android) and allows the smartphone to connect to many Bluetooth health devices from different vendors would increase the efficiency of health care applications and provide users with more choices.

**Figure 5:**
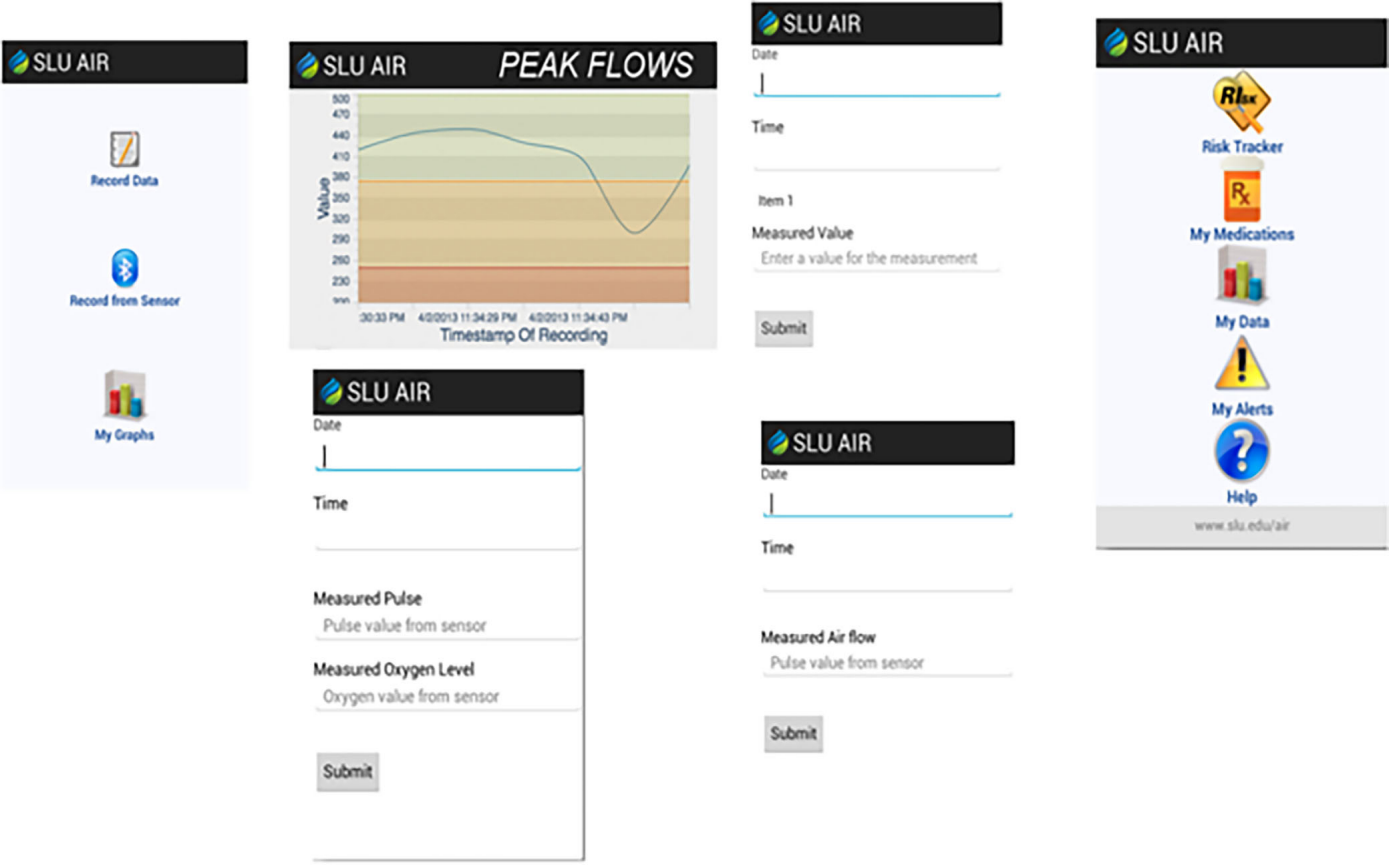
Screenshots for the SLU AIR application

**Figure 6:**
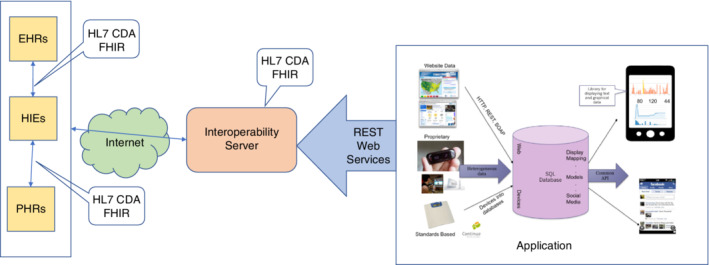
SLU AIR System architecture

SLU AIR, a prototype self‐management asthma application for Android smartphones, was developed to enable further patient evaluation and testing. This includes integration with a Bluetooth enabled peak flow meter and the ability to exchange this device data with the Microsoft Personal Health Care Record service. It should be noted that Microsoft's Personal Health Care Record service is being discontinued in November 2019.^63^ Microsoft's HealthVault was chosen for several reasons: well‐documented API to exchange data^5^; free to use^30^; and does not require access to the EHR of any organization.^22^ Excluding interoperability with traditional EHRs, the SLU AIR prototype has met some of the requirements of the focus groups. Screenshots for the working application are shown in Figure 5, and the complete overall system architecture is shown in Figure 6.

## 4 DISCUSSION

This prototype is developed to run only under the Android smart phone operating system because Android is a more open development environment in several contexts. First, Android provides a defined interface into their Bluetooth communication stack. Apple iOS did not support this feature at the time prototype development started. The prototype does not communicate with traditional EHRs such as EPIC because of political complexity and cost. To enhance interoperability, a new infrastructure and communication interface would need to be created, as well as the development of the new interface into the existing EHR applications.

In addition to the application itself, infrastructure to support the application was also created. Today, the complexity of exchanging health care information with providers requires an interoperable server. This server can communicate with several existing and emerging standards to provide a uniform interface to the smartphone application. SLU AIR's server currently exchanges data with Microsoft's HealthVault PHR. The connection to HealthVault illustrated that the prototype is well defined and can exchange data over the Internet. Future versions could communicate with EHRs and Health Information Exchanges (HIEs) and will allow data to be pulled with the emerging Fast Healthcare Interoperability Resource (FHIR) standard. The RESTful web services API using FHIR resources are likely to become the standardized methods for data exchange.^64^ These resources are suited for mobile applications and allow direct communication with any health information application that supports RESTful FHIR. Furthermore, the interoperability server will also support older CDA/HL7v3 data exchange to support legacy systems. The smartphone will communicate with the server under the framework currently being defined by the HL7 group and promoted by the Office of the National Coordinator.^65^

This work contributes and advances the use of UCD in mobile health applications in several ways:

1. Unlike breathe^28^ or other studies,^42^, ^66^ utilizing UCD for a mobile asthma application SLU AIR is designed around a learn/sense/suggest architecture. Breathe has several features that need AI technology such as identifying triggers and suggesting to patients which triggers to avoid and when to avoid them. However, breathe is not designed with learning, sensing, and tailored suggesting as its underlaying architecture.
2. Morita does an excellent job of applying UCD to directly develop breathe. Our methodology differs in that we apply UCD to develop a set of attributes for SLU AIR, then map these requirements into a generalized infrastructure to design a learning health system for any chronic condition. Using this architecture, we designed SLU AIR. This generalized infrastructure is more scalable and extendable to other clinical conditions.
3. Integration of automatic peak flow metrics from patient to the EHR to improve the quality of data and make this data collection easy for the patient.

One limitation of our study is no user testing of the functional prototype.

## 5 CONCLUSION

Smartphone applications can be a useful tool to help manage chronic illnesses, such as asthma. However, a UCD approach must be taken during application development in order to ensure that the needs of patients and providers are being met, while intelligent learning systems will ensure applications are continuously being tailored and improving usability for each user. Very few examples of these features were found in the literature, reflecting that applications are lacking features desired by patients and health care providers. Patients and providers want an interactive smartphone app for asthma that truly helps them maintain better health with fewer interruptions in their life from asthma exacerbations. With a focus on the patients, providers, and intelligent learning, SLU AIR attempts to be that product for asthma patients.

## CONFLICT OF INTEREST

All authors declare that they have no conflicts of interest.
